# Overexpression of *CsLEA11*, a Y_3_SK_2_-type dehydrin gene from cucumber (*Cucumis sativus*), enhances tolerance to heat and cold in *Escherichia coli*

**DOI:** 10.1186/s13568-017-0483-1

**Published:** 2017-09-29

**Authors:** Yong Zhou, Peng He, Yaping Xu, Qiang Liu, Yingui Yang, Shiqiang Liu

**Affiliations:** 1 Department of Biochemistry and Molecular Biology, College of Science, Jiangxi Agricultural University, Nanchang, Jiangxi China; 2 Department of Horticulture, College of Agronomy, Jiangxi Agricultural University, Nanchang, Jiangxi China; 3 Economic Specialty Station of Wucheng District, Jinhua, Zhejiang China

**Keywords:** *Cucumis sativus*, Dehydrin (DHN), Intrinsically disordered protein (IDP), Expression analysis, Abiotic stress

## Abstract

As the group II LEA (late embryogenesis abundant) proteins, dehydrins (DHNs) play an important role in plant growth and development, as well as in response to abiotic or biotic stress challenges. In this study, a *DHN* gene named *CsLEA11* was identified and characterized from *Cucumis sativus*. Sequence analysis of CsLEA11 showed that it is a Y_3_SK_2_-type DHN protein rich in hydrophilic amino acids. Expression analyses revealed that the transcription of *CsLEA11* could be significantly induced by heat and cold stress. The recombinant plasmid was transformed into *Escherichia coli* BL21 and isopropy-β-d-thiogalactoside (IPTG) was used to induce recombinant *E. coli* to express *CsLEA11* gene. Overexpression of *CsLEA11* in *E. coli* enhanced cell viability and conferred tolerance to heat and cold stress. Furthermore, CsLEA11 protein could protect the activity of lactate dehydrogenase (LDH) under heat stress. Taken together, our data demonstrate that *CsLEA11* might function in tolerance of cucumber to heat and cold stress.

**Electronic supplementary material:**

The online version of this article (doi:10.1186/s13568-017-0483-1) contains supplementary material, which is available to authorized users.

## Introduction

Temperature is one of the major factors affecting plant growth and productive capacity (Guo et al. 2017). Higher plants have evolved a variety of strategies for continuous changes to deal with the unfavorable stresses, such as heat and cold. For example, LEA (late embryogenesis abundant) proteins are synthesized in large amounts during the late stages of seed development, enabling the maturing seeds to acquire desiccation tolerance, which plays an important role in the adaptation of plants to abiotic stresses (Hu et al. 2016; Tang et al. 2016; Tolleter et al. 2007).

As the group II LEA, dehydrins (DHNs) are highly hydrophilic proteins, and contain three conserved motifs named as K-, Y-, and S-segments. Based on the numbers of Y-, S- and K-segments, DHNs can be subdivided into five major types, including Y_n_SK_n_, K_n_, SK_n_, Y_n_K_n_, and K_n_S (Close 1996; Lv et al. 2017). Since *DHN* genes are expressed in various plant tissues including roots, stems, leaves, flowers, fruits and seeds, different types of *DHN* genes may be involved in response to diverse abiotic stresses including heat and cold (Abedini et al. 2017; Charfeddine et al. 2017; Jing et al. 2016; Verma et al. 2017). For example, the expression of three *DHN* genes was detected in sugarcane under heat stress, and their expression is independent of the changes in the water relations of leaves (Wahid and Close 2007). Both Grapevine *DHN1* and *DHN2* are induced by heat and cold, but their expression profiles differ appreciably from each other (Yang et al. 2012). In Tifway, *CdDHN4* is greatly up-regulated by heat and cold treatments, and its expression under cold treatment is significantly higher than that under heat treatment (Lv et al. 2017).

There are increasing evidences showing that there are extensive correlative links between DHNs and tolerance to heat or cold stress in organisms. For example, wheat DHN-5 is able to protect enzyme activities in vitro from adverse effects induced by heating (Brini et al. 2010). Wheat WZY2 can act as a protectant to increase *Escherichia coli* viability, protect lactate dehydrogenase (LDH) activity and inhibit protein aggregation during temperature variation including heat and cold (Yang et al. 2015). A number of studies have shown that the cold stress tolerance was enhanced by overexpression of different *DHNs* in various plant species, such as *Arabidopsis* (Aguayo et al. 2016; Ochoa-Alfaro et al. 2012; Peng et al. 2008), tobacco (Guo et al. 2017; Hill et al. 2016; Liu et al. 2014; Xing et al. 2011), strawberry (Houde et al. 2004), cucumber (Yin et al. 2006), and tomato (Liu et al. 2015), suggesting their possible functions in cold stress. Besides, loss of function of some *DHN* genes can also decrease the cold stress tolerance of transgenic plants. For example, silencing of either *CaDHN1* or *CaDHN3* in pepper resulted in obviously lower resistance to cold stress compared with the control (Chen et al. 2015; Jing et al. 2016).

A previous study examined the *LEA* gene family in cucumber and found that one of *DHN* genes named *CsLEA54* might facilitate the adaptation of cucumber to drought stress (Altunoglu et al. 2016). In this study, we isolated another *DHN* gene, *CsLEA11*, and examined its expression under heat and cold stress. Recombinant CsLEA11 protein was expressed and purified from *E. coli*. *E. coli* overexpressing *CsLEA11* showed significantly improved tolerance to heat and cold stress. The effect of CsLEA11 protein on LDH activity under heat stress was also examined in vitro.

## Materials and methods

### Plant materials and treatments

Two-week-old cucumber (*Cucumis sativus* L. cv. Chinese long No. 9930) seedlings were grown in a growth chamber under a long-day (16 h of light/8 h of dark) photoperiod at 400 µmol m^−2^ s^−1^ light intensity and a relative humidity of 60–70%. For heat and cold treatment, cucumber seedlings in the growth chamber were subjected to 42 and 4 °C under normal light conditions. The leaf tissues were harvested from seedling samples at 0, 3, 6, 12 and 24 h, frozen in liquid nitrogen immediately, and stored at −80 °C until use.

### Isolation and sequence analysis of CsLEA11

Total RNA was extracted from leaves using Trizol reagent according to the manufacturer’s instructions (Tiangen, China). The quality of the extracted RNA was examined by agarose gel electrophoresis. The first strand cDNA was synthesized using the Superscript™ III RNase H-Reverse Transcriptase kit (Invitrogen, USA). The full-length cDNA sequence of *CsLEA11* was amplified using gene-specific primers designed according to the cucumber genome initiative (CuGI, http://cucumber.genomics.org.cn, Gene ID: Csa020767), and NCBI database (GenBank Accession Number: XM_004150027.2). The PCR product was cloned into the pGEM-T (Promega, USA) and subsequently sequenced.

The theoretical pI, MW and the grand average of hydropathy index (GRAVY) of the deduced CsLEA11 protein were predicted by the ProtParam program (http://web.expasy.org/protparam). The prediction of protein secondary structure and intrinsic disorder in the deduced CsLEA11 protein was performed by the PHYRE2 protein fold recognition server (http://www.sbg.bio.ic.ac.uk/phyre2/html/page.cgi?id=index). The multiple sequence alignments of CsLEA11 and other dehydrin proteins were performed with Clustal W software (Larkin et al. 2007). A phylogenetic tree was then constructed with the MEGA 5.0 software by the neighbor-joining method with 1000 bootstrap replicates (Tamura et al. 2007).

### Quantitative real-time PCR (qRT-PCR)

Quantitative real-time PCR (qRT-PCR) was performed with SYBR premix Ex Taq kit (TaKaRa, Japan) according to the manufacturer’s instructions. Amplification was carried out on the LightCycler 480 System (Bio-Rad, USA). Each PCR mixture was composed of 10 μL of 1× SYBR premix Ex Taq, 0.1 μL of reverse and forward primers (0.5 μM each) and 2 μL of cDNA, and was added up to 20 μL with nuclease-free water. The procedure was implemented as follows: 94 °C for 5 min, followed by 40 cycles of 94 °C for 5 s, 55 °C for 10 s, and 72 °C for 15 s. The relative expression level was calculated using the 2^−ΔΔCT^ method (Livak and Schmittgen 2001), and the C_T_ values were obtained from three independent biological replicates. An endogenous *actin* gene (*CsAct3*, GenBank Accession Number: DQ115883) was used as an internal reference (Wan et al. 2010). The primers are listed in Table 1.

**Table 1:** Sequences of primers used for qRT-PCR analysis

Primer	Sense (5′ → 3′)	Antisense (5′ → 3′)
CsLEA11	CGAGCAGTTCCAGCTCTA	CTTCCGGTTAACTTCTCCTT
CsActin	GAATCCAGCACGATACCA	TCAACCCAAAGGCTAACA

### Induction and expression of CsLEA11 protein

To amplify the coding sequence of *CsLEA11*, the primers 5′-aaaaGGATCCATGGCGAATGTACGCGATGAG-3′ (*Bam*H I restriction site underlined) and 5′-aaaaAAGCTTATGATGGTGGCCAGGTAATTTC-3′ (*Hin*d III restriction site underlined) were designed. PCR amplification was conducted using the following protocol: pre-denaturation for 5 min at 94 °C, 30 cycles at 94 °C for 30 s, 58 °C for 30 s, 72 °C for 30 s, followed by 1 cycle at 72 °C for 10 min. Then the PCR product was cloned into pET32a, the resulting construct (pET32a-CsLEA11) was confirmed by sequencing and transformed into the *E. coli* host strain BL21 (DE3). The cells with pET32a-CsLEA11 and pET32a plasmids were induced with IPTG (1 mM) at 37 °C for 4 h and analyzed by SDS-PAGE as previously described (Zhou et al. 2017).

### Assay of heat and cold stress tolerance of *E. coli* transformants

Stress tolerance assays were performed to detect the effect of CsLEA11 on *E. coli* viability under heat and cold stress conditions. IPTG induction and cell cultures were prepared as described above until the optical density (OD_600_) was adjusted to 0.6. Subsequently, 2 mL of the IPTG-induced transformant strains were inoculated into 50 mL fresh liquid LB with 1 mM IPTG and 50 μg/mL ampicillin, and the initial concentration was adjusted to the same OD_600_ for each stress. *E. coli* cells containing pET32a-CsLEA11 and pET32a (as a control) were named as BL/CsLEA11 and BL/pET32a, respectively. For heat stress, 1 mL of culture medium was incubated at 50 °C for different periods of time (10, 20, 30, 40 and 50 min), and then 100 μL of dilution (1:10) was spread onto LB agar plates with 1 mM IPTG and 50 μg/mL ampicillin. The cold stress tolerance assay of *E. coli* transformants was performed using the method of a previous study (Hu et al. 2016). In brief, a 1-mL aliquot of the BL/CsLEA11 and BL/pET32a cells was frozen in liquid nitrogen for 1 min, and then thawed for 15 min at 37 °C, which made a cycle. The aliquots were taken after 3, 6, 9, 12 and 15 cycles, respectively, and then 100 µL of dilution (1:10) was spread onto LB agar plates with 1 mM IPTG and 50 μg/mL ampicillin. After incubation overnight at 37 °C, the cell viability was calculated by counting the number of colony-forming units as previously described (Zhou et al. 2017).

### LDH activity assay

The LDH enzyme (EC1.1.1.27) from muscle lactate (Sigma, USA) was diluted to a final concentration of 1 mg/mL in phosphate buffer saline (PBS). The thermal treatment and measurement of LDH activity were carried out according to the previously published method (Zhou et al. 2017). The purified CsLEA11 protein was added to LDH at the mass ratio of 1:5, and PBS and BSA were used as negative and positive controls, respectively. Each experiment was performed in triplicate.

### Statistical analysis

All statistical analyses were performed using the Statistical Package for Social Sciences (SPSS 19.0) with Student’s t test in a two-tailed analysis, and statistical significance was set at *P < 0.05 and **P < 0.01.

## Results

### Clone and sequence analysis of *CsLEA11*

A 846-bp cDNA fragment containing a 489-bp ORF was amplified from cucumber leaves by semi-quantitative RT-PCR. A sequence of 1614 bp was obtained from cucumber genomic sequence, which consists of two exons and one intron. This gene encodes a protein of 162 amino acids with a predicted theoretical isoelectric point and molecular weight of 8.11 and 17.47 kDa, respectively. According to a previous study, this gene was named as *CsLEA11* (Altunoglu et al. 2016).

**Fig. 1:**
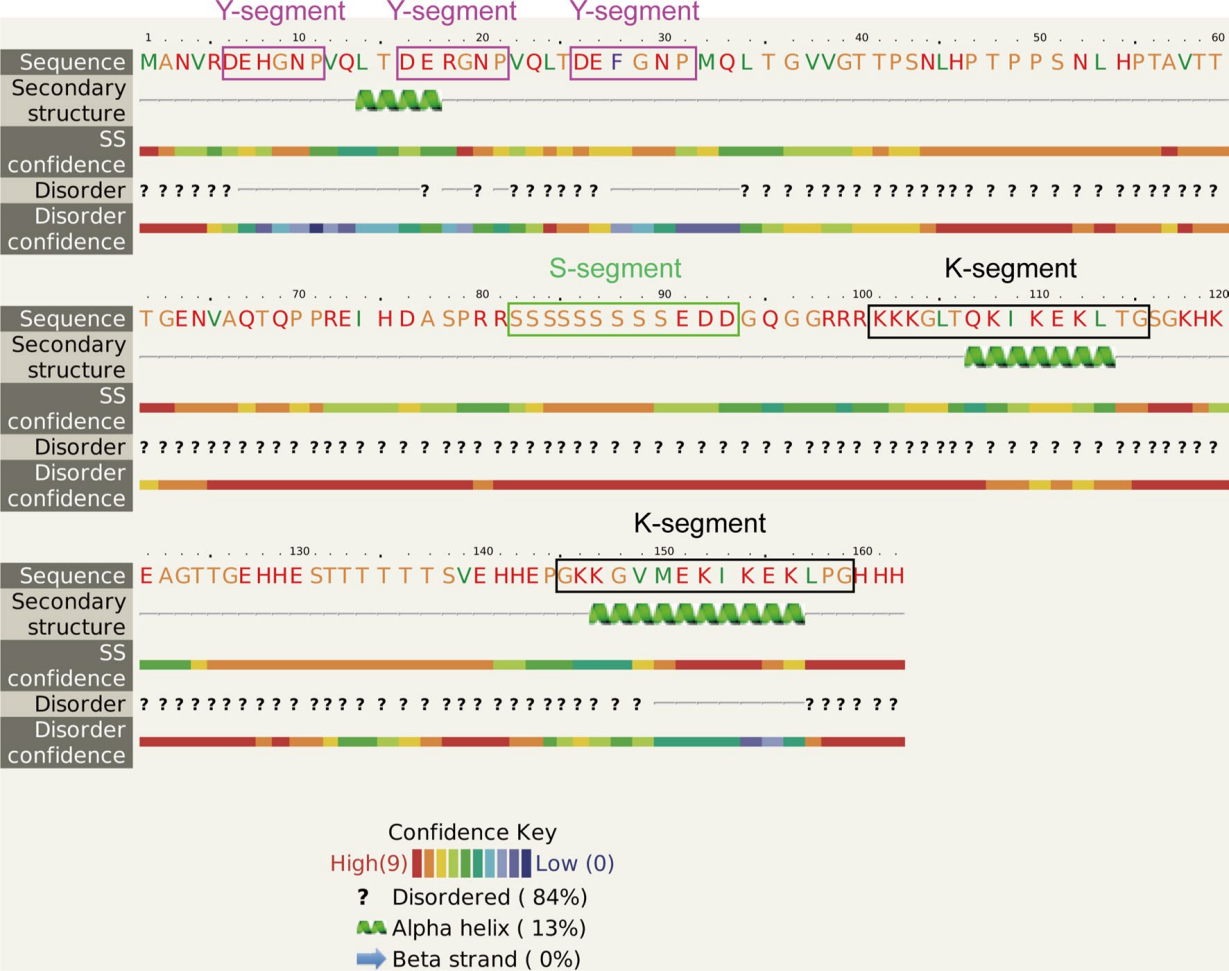
Deduced amino acid sequence of CsLEA11. The Y-, S- and K-segments are boxed by purple, green and black, respectively

The CsLEA11 protein contains three Y-segments, one S-segment, and two K-segments. Therefore, it was classified as a Y_3_SK_2_-type DHN (Fig. 1). The CsLEA11 protein was rich in hydrophilic amino acids such as Thr (13.0%), Gly (10.5%), Ser (9.3%), Glu (8.6%), Lys (8.0%), and His (7.4%), but was lack of the hydrophobic amino acids Cys and Trp. The grand average of hydropathicity (GRAVY) of the deduced CsLEA11 protein was −1.230, indicating that it is strongly hydrophilic. In addition, in silico prediction with a protein secondary structure and intrinsic disorder tool (PHYRE2) suggested that over 84% of the amino acid residues of CsLEA11 were disordered, and CsLEA11 harbored 13% of alpha helices, which are mainly present in the two K-segments (Fig. 1). These results suggested that CsLEA11 is an intrinsically disordered protein (IDP).

**Fig. 2:**
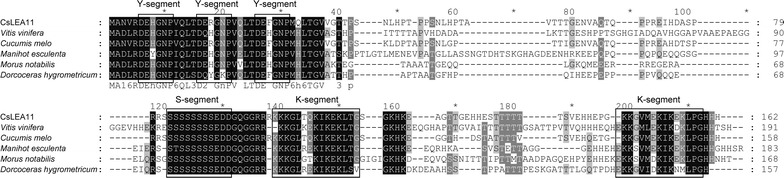
Sequence alignment of CsLEA11 protein with homologs from other plant species performed with the NCBI database. Accession Numbers are as follows: *Vitis vinifera* (XP_002283605.1), *Cucumis melo* (XP_008460975.1), *Manihot esculenta* (AGC51773.1), *Morus notabilis* (XP_010105346.1), and *Dorcoceras hygrometricum* (KZV53366.1). The multiple amino acid sequence alignment reveals the common features of CsLEA11 and other DHNs, such as the Y-, S- and K-segments, which are boxed

Sequence alignment of the deduced amino acid sequence of CsLEA11 with that of other DHNs indicated that it was homologous to sequences from *Cucumis melo* (XP_008460975.1, 86.5% identity), *Dorcoceras hygrometricum* (KZV53366.1, 53.8% identity), *Morus notabilis* (XP_010105346.1, 53.1% identity), *Vitis vinifera* (VvDHN4, XP_002283605.1, 49.7% identity), and *Manihot esculenta* (AGC51773.1, 48.9% identity). Sequence alignment results of CsLEA11 and these closely related DHNs are shown in Fig. 2, which further confirmed that CsLEA11 is a Y_3_SK_2_-type DHN protein. In addition, both the Y- and K-segments were slightly different from the reported consensus sequences (DEYGNP and EKKGIMDKIKEKLPG, respectively) with a few conservative substitutions (Fig. 2).

**Fig. 3:**
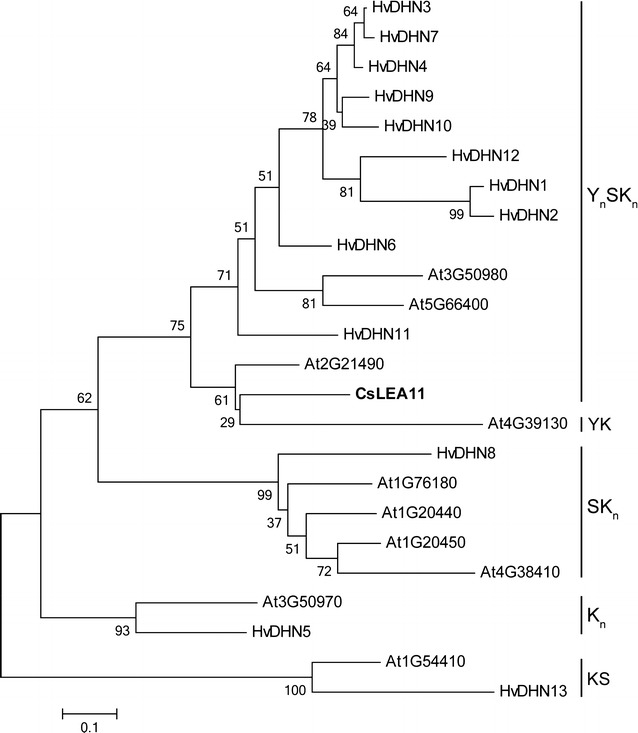
Phylogenetic tree of amino acid sequences from CsLEA11 and DHNs from other plant species including *Arabidopsis* and barley. The tree was constructed with MEGA 5.0 program using neighbor-joining method with 1000 bootstrap replicates. The amino acid sequences used to generate the phylogenic tree were listed in Additional file 1: Table S1

In order to evaluate the molecular evolutionary relationships between CsLEA11 and DHN proteins from other plant species, a phylogenetic tree was constructed (Fig. 3). The results showed that these DHNs could be divided into five groups: Y_n_SK_n_, YK, SK_n_, K_n_, and KS. CsLEA11 shares high genetic homology with two *Arabidopsis* genes At4g39130 and At2g21490, which belong to the YK and Y_n_SK_n_ group, respectively (Fig. 3).

### Transcript analysis of *CsLEA11* under heat and cold stress

**Fig. 4:**
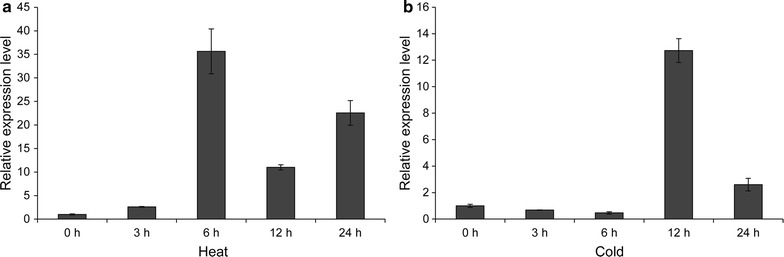
qRT-PCR analysis of *CsLEA11* expression in the leaves of cucumber plants under heat (**a**) and cold (**b**) stress conditions. The results are the mean ± SD of three independent experiments

To determine the expression of *CsLEA11* under heat and cold stress, the transcript of *CsLEA11* was analyzed by qRT-PCR. Under heat stress, the transcript level of *CsLEA11* first increased with time and peaked at 6 h (35.6-fold), then decreased to 11.0-fold at 12 h, and was finally detected to be 22.6-fold at 24 h. However, contrary to the case of heat stress treatment, the transcripts of *CsLEA11* under cold stress decreased with time until 6 h, reached the maximum level at 12 h (12.7-fold), and then decreased at 24 h (Fig. 4b).

### Induction of fusion protein of CsLEA11 in *E. coli* cells

**Fig. 5:**
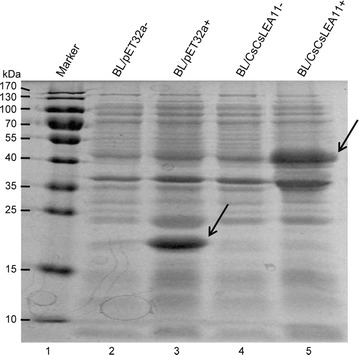
SDS-PAGE analysis of TrxA-CsLEA11 fusion protein in *E. coli* cells before and after IPTG induction. Lane 1, marker; lane 2, BL/pET32a without IPTG induction; lane 3, BL/pET32a after IPTG induction; lane 4, BL/CsLEA11 without IPTG induction; lane 5, BL/CsLEA11 after IPTG induction. The arrows indicate the protein band corresponding to the recombinant

The CsLEA11 protein was expressed in *E. coli* BL21 (DE3) and analyzed by SDS-PAGE. As shown in Fig. 5, compared with non-induced cells, cells of *E. coli* harboring pET32a vector (BL/pET32a) produced TrxA protein of 21.1 kDa, which is consistent with the results of previous studies (Hu et al. 2016; Zhou et al. 2017). The fusion protein presented a molecular weight of about 40 kDa, which is identical with the expected size (Fig. 5, lane 5), and this band was not detected in non-induced BL/CsLEA11 cells (Fig. 5, lane 4), indicating that the TrxA-CsLEA11 fusion protein was correctly expressed in recombinant *E. coli*.

### CsLEA11 overexpression enhanced *E. coli* growth under heat and cold stress

**Fig. 6:**
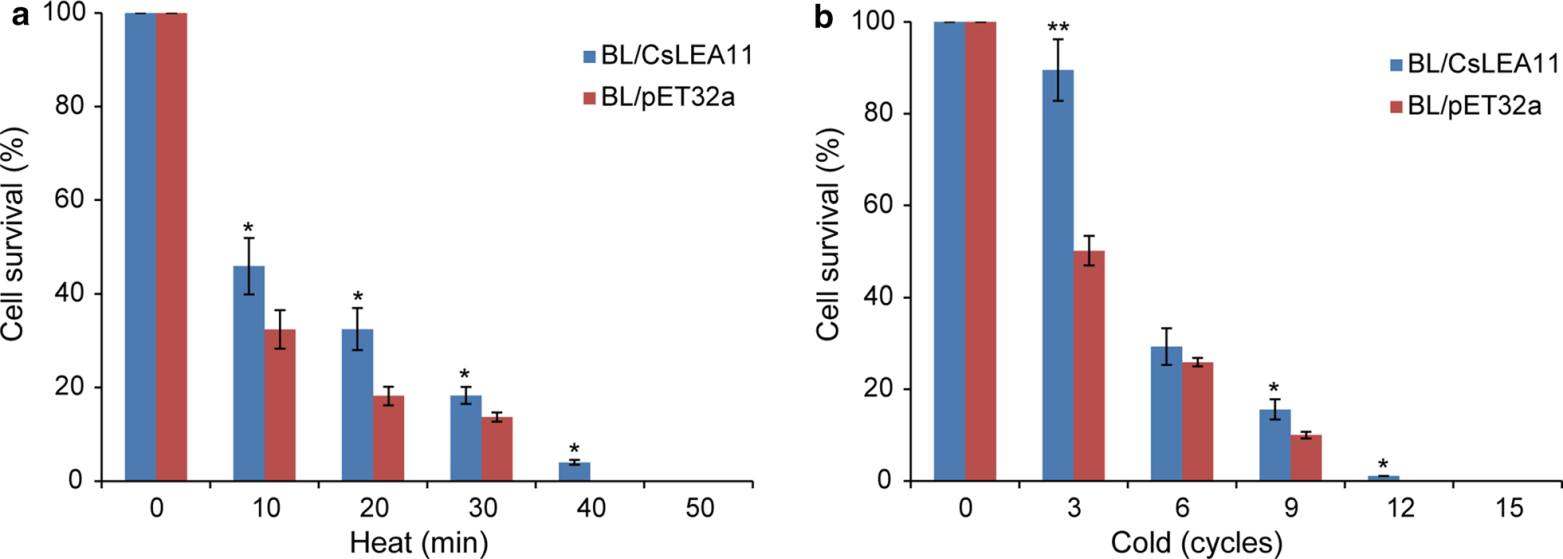
Growth performance of BL/CsLEA11 and BL/pET32a cells under heat (**a**) and cold treatments (**b**). Error bars represent standard deviations generated from three replicate trials

To assess the effect of CsLEA11 on the growth of *E. coli* recombinants under heat and cold stress, BL/CsLEA11 and BL/pET32a cells were subjected to heat and cold treatment, respectively. Under normal conditions, the growth pattern of BL/CsLEA11 cells was similar to that of BL/pET32a (Fig. 6), implying that the expression of CsLEA11 protein does not affect the growth of the *E. coli* recombinant. Under heat treatment, the cell viability of BL/CsLEA11 and BL/pET32a cells decreased rapidly. However, the cells of BL/CsLEA11 showed greater proliferation than those of BL/pET32a at the time points of 10, 20, 30 and 40 min, and both of types of cells died at 50 min (Fig. 6a). Similar results were observed under cold treatment (Fig. 6b). After 3, 6, 9 and 12 freeze–thaw cycles, the cell viability of BL/CsLEA11 cells was about 89.5, 29.3, 15.6, and 1.1%, respectively, whereas that of the BL/pET32a cells was 50.1, 25.9, 10, and 0%, respectively. Similarly, both BL/CsLEA11 and BL/pET32a cells died after 15 freeze–thaw cycles (Fig. 6b). These results indicated that CsLEA11 confers tolerance to heat and cold stress in *E. coli*.

### CsLEA11 protected LDH enzyme activity under heat stress

**Fig. 7:**
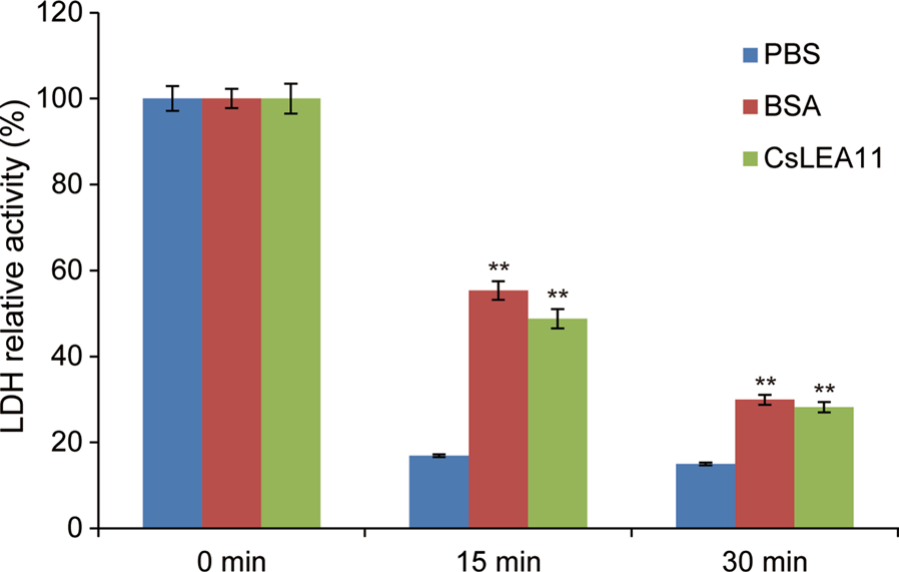
Protection of LDH activity by CsLEA11 protein from inactivation under heat stress. The relative activity of LDH during heating at 65 °C for 15 and 30 min. The LDH activity and standard deviations are based on three independent experiments

LDH activity was used as a marker to evaluate the protective effect of CsLEA11 against heat stress. As shown in Fig. 7, the activity of LDH sharply decreased after treatment at 65 °C for 15 min or 30 min with or without CsLEA11. In contrast to that of negative control (PBS), the loss of LDH activity in the presence of CsLEA11 and positive control (BSA) was much slower. After the treatment at 65 °C for 15 min, the LDH activity decreased to 16.9, 55.4 and 48.8% of its initial value in the presence of PBS, BSA and CsLEA11, respectively. Additionally, LDH activity with PBS decreased to 15.0%, whereas it remained to be 30.0 and 28.2% of its initial value upon incubation for 30 min in the presence of CsLEA11 and BSA, respectively. Under heat stress, although the LDH activity in the presence of CsLEA11 also decreased, it was still 2.9- and 1.9-fold that in the presence of PBS at 15 and 30 min, respectively (Fig. 7). These results revealed that just like BSA, CsLEA11 can also efficiently protect LDH activity under heat stress.

## Discussion

Over the past few decades, numerous *DHN* genes have been identified in various plant species, and were demonstrated to play important roles in abiotic stress tolerance (Banerjee and Roychoudhury 2016; Liu et al. 2017). In this study, a *DHN* gene named *CsLEA11* was for the first time isolated and characterized from *C. sativus*. Sequence alignment and phylogenetic analysis indicate that CsLEA11 belongs to the Y_3_SK_2_ group. Gene expression analyses are important for the revealing of gene functions. There are increasing evidences showing that *DHN* genes are regulated by various abiotic stresses including heat and cold (Lv et al. 2017; Wahid and Close 2007; Yang et al. 2012). In this study, the transcription of *CsLEA11* was induced by heat and cold treatments (Fig. 4), implying a positive correlation between *CsLEA11* and tolerance to heat or cold stress.

Heterologous expression is an easy and effective method to investigate the function of a protein in vivo with less time, energy and resources (Jabeen et al. 2017; Wang et al. 2017). Several recent studies have investigated the overexpression of *DHN* genes in *E. coli* cells to reveal their possible functions under stress conditions. For example, heterologous expression of CarDHN from *Cerastium arcticum* in *E. coli* resulted in increased intrinsic tolerance to cold stress (Kim et al. 2013). Overexpression of a dehydrin-like protein gene *AmCIP* from *Ammopiptanthus mongolicus* conferred cold stress tolerance to *E. coli* (Shi et al. 2016). A recent study reported that the *E. coli* cells expressing some *Pinus tabuliformis* DHN proteins showed enhanced viability and tolerance under salt and heat stress (Gao and Lan 2016). Another report also revealed that overexpression of *Dofdehydrin*-*1* from *Dendrobium officinale* enhanced *E. coli* viability under salt and heat stress (Ling et al. 2016). Very recently, Bao et al. (2017) reported that the expression of nearly all of the *Prunus mume* dehydrins improved osmotic and cold resistance of the recombinant *E. coli*. In this study, recombinant *E. coli* cells overexpressing CsLEA11 showed higher cell viability than the control under heat and cold stress (Fig. 6), which is in agreement with previous results, implying that CsLEA11 might participate in adaptive responses to heat and cold stress.

A number of studies have demonstrated that many DHNs lack Cys and Trp residues and are rich of charged and polar amino acids (Close 1996; Jing et al. 2016; Yang et al. 2015). CsLEA11 also has these properties, which make it unstructured and highly hydrophilic, suggesting that it is an IDP (Fig. 1) (Dyson and Wright 2005). Because IDPs are lack of a unique and well-defined protein structure, they can function in the protection of other cellular proteins and stabilization of plant outer and organellar membranes under stress conditions (Jha et al. 2012; Rahman et al. 2010; Uversky and Dunker 2010). It has been shown that some DHNs can prevent the inactivation of malate dehydrogenase (MDH), LDH, alcohol dehydrogenase (ADH), catalase or citrate synthase (CS) enzymes under diverse stress conditions in vitro (Drira et al. 2013; Kovacs et al. 2008; Shi et al. 2016; Yang et al. 2015). For example, wheat DHN-5 is able to protect the activities of LDH, β-glucosidase and glucose oxidase from the adverse effects induced by cold and heat (Brini et al. 2010; Drira et al. 2013). *Arabidopsis* ERD10 (At1G20450) and ERD14 (At1G76180) were found to prevent the heat-induced aggregation and denaturation of lysozyme, alcohol dehydrogenase, firefly luciferase and citrate synthase (Kovacs et al. 2008). Another *Arabidopsis* dehydrin AtHIRD11 (At1G54410) can even reactivate the LDH inhibited by Cu^2+^ through removing Cu^2+^ from the active site (Hara et al. 2016). In the present study, CsLEA11 could protect the activity of LDH from heat-induced inactivation (Fig. 7). Similar results were also observed for DHN-5 (Brini et al. 2010) and WZY2 from wheat (Yang et al. 2015), and SbDhn2 from sorghum (Halder et al. 2016). Hence, CsLEA11 can protect *E. coli* exposed to heat and cold stress, probably via protection of some stress-related proteins from inactivation under stress conditions.

In conclusion, we have cloned and characterized a *DHN* gene named *CsLEA11* from *C. sativus*. The expression of *CsLEA11* is observably up-regulated by heat and cold treatment, and overexpression of *CsLEA11* improves the growth performance of *E. coli* cells under heat and cold stress conditions. In addition, CsLEA11 protein can protect LDH activity in vitro from heat-induced inactivation. Our results show that *CsLEA11* may have a potential in genetic improvement for plants to adapt to heat and cold stress.

## Additional file

**Additional file 1: Table S1.** DHN sequences used for phylogenetic tree analysis.

## Abbreviations

LEA: late embryogenesis abundant
DHN: dehydrin
LDH: lactate dehydrogenase
*E. coli*: *Escherichia coli*
qRT-PCR: quantitative real-time PCR
PBS: phosphate buffer saline
IDP: intrinsically disordered protein
