# Supplementary material for: Overexpression of CsLEA11, a Y3SK2-type dehydrin gene from cucumber (Cucumis sativus), enhances tolerance to heat and cold in Escherichia coli
Source: AMB Express. 2017 Sep 29;7:182. doi: 10.1186/s13568-017-0483-1 (PMC5622017; doi:10.1186/s13568-017-0483-1)
Supplement: Supplementary file 1 — Additional file 1: Table S1. DHN sequences used for phylogenetic tree analysis. [file 13568_2017_483_MOESM1_ESM.doc]

**Table S1.** DHN sequences used for phylogenetic tree analysis.

| **Name** | **Protein ID** | **Type** | **Species** |
| --- | --- | --- | --- |
| CsLEA11 | Csa020767 | YnSKn | *Cucumis sativus* |
|  |  |  |  |
| At1G20440 | At1G20440 | SKn | *Arabidopsis thaliana* |
| At1G20450 | At1G20450 | SKn | *Arabidopsis thaliana* |
| At1G54410 | At1G54410 | KS | *Arabidopsis thaliana* |
| At1G76180 | At1G76180 | SKn | *Arabidopsis thaliana* |
| At2G21490 | At2G21490 | YnSKn | *Arabidopsis thaliana* |
| At3G50970 | At3G50970 | Kn | *Arabidopsis thaliana* |
| At3G50980 | At3G50980 | YnSKn | *Arabidopsis thaliana* |
| At4G38410 | At4G38410 | SKn | *Arabidopsis thaliana* |
| At4G39130 | At4G39130 | YK | *Arabidopsis thaliana* |
| At5G66400 | At5G66400 | YnSKn | *Arabidopsis thaliana* |
|  |  |  |  |
| HvDHN1 | AAD02253.1 | YnSKn | *Hordeum vulgare* |
| HvDHN2 | AAD02254 | YnSKn | *Hordeum vulgare* |
| HvDHN3 | AAD02255 | YnSKn | *Hordeum vulgare* |
| HvDHN4 | AAD02256 | YnSKn | *Hordeum vulgare* |
| HvDHN5 | AAD02262 | Kn | *Hordeum vulgare* |
| HvDHN6 | AAD02257 | YnSKn | *Hordeum vulgare* |
| HvDHN7 | AAD02258 | YnSKn | *Hordeum vulgare* |
| HvDHN8 | AAD02259 | SKn | *Hordeum vulgare* |
| HvDHN9 | AAD02260 | YnSKn | *Hordeum vulgare* |
| HvDHN10 | AAD02261 | YnSKn | *Hordeum vulgare* |
| HvDHN11 | AAD02252 | YnSKn | *Hordeum vulgare* |
| HvDHN12 | AAD38400 | YnSKn | *Hordeum vulgare* |
| HvDHN13 | AAT81473 | KS | *Hordeum vulgare* |
